# A novel internet sampling for HIV surveillance: feasibility of self-sampling and preparation of DBS for delivery detection of HIV total nucleic acid and complementarity to sentinel surveillance

**DOI:** 10.1186/s12879-023-08456-w

**Published:** 2023-08-04

**Authors:** Xueli Su, Dongyan Xia, Yanming Sun, Yinxiao Hao, Guowu Liu, Chun Huang, Hongyan Lu

**Affiliations:** Department of AIDS/STDs Control and Prevention, Beijing Center for Disease Control and Prevention, Beijing, China

**Keywords:** Internet plus, Self-sampling, DBS, HIV total nucleic acid, Sentinel surveillance

## Abstract

**Background:**

To propose a new mode of HIV test and surveillance among population of men who have sex with men (MSM): Internet-based Self-sampling at home plus Laboratory testing of HIV total nucleic acid (TNA) in dried blood spot (DBS) (ISL of DBS TNA). Feasibility of ISL of DBS TNA was studied. Characteristics of the new mode and that of conventional surveillance mode at HIV voluntary counseling and testing clinic (VCT) were compared.

**Methods:**

A non-governmental organization (NGO) published the recruitment information on the WeChat public account. MSM filled in the questionnaire online, applied for self-sampling service package, and mailed the self made DBS to professional laboratory. The laboratory performed HIV TNA test and submitted the test results to online platform. Participants queried test results online with their unique ID. Center for Disease Control and Prevention (CDC) followed up participants with positive nucleic acid results using IDs and contact information. Rates were compared by using the Chi-Square test or Fisher's exact test.

**Results:**

Four hundred twenty-three questionnaires were completed. 423 self-sampling service packages were sent out and 340 DBSs were returned to professional laboratory within one month with qualified rate of sampling as high as 95.0% (323/340). Seven samples were found to be TNA positive. Comparing ISL of DBS TNA with sentinel surveillance, it was found that there was a significant difference in the composition ratio of the two modes of surveillance population (*P* < 0.05). HIV prevalence of ISL of DBS as 2.17% was significantly lower than sentinel site as 8.96% (*χ*^*2*^ = 14.953, *P* = 0.000 < 0.05).

**Conclusions:**

ISL of DBS TNA proposed is feasible and has a high self-sampling qualification rate, good confidentiality. It is an effective supplement to routine sentinel surveillance and has important promotion value.

## Background

The AIDS epidemic in China is still very severe. According to Chinese Center for Disease Control and Prevention, the survival number of HIV-infected patients was estimated at 850,000 by the end of September 2018. However, 30% of the infected persons are still undetected [1]. Low detection rate is the key bottleneck to contain the AIDS epidemic in China. High-risk sexual behavior of infected persons led to the vast majority of new HIV transmission, producing a new round of HIV infection cases. Studies show that 50% of new infections come from the second generation transmission of early infected patients [2–4]. Acute infections are estimated to be 26 times more infectious than asymptomatic infections [5, 6]. Initial viral load is up to 10^6^-10^7^cp / ml, which is highly infectious. The HIV infected by MSM is mainly a CXCR4 tropical strain and is highly pathogenic. The average incubation period is only 4–5 years, significantly shorter than the 8–10 years incubation period of other strains [7]. The infections need to be detected and treated as soon as possible. However, many infected people failed to receive HIV test timely, but were found late in the hospital due to various complications when the immune system was seriously damaged, resulting in high mortality rate. Sexual transmission is the main route of AIDS transmission in China. Heterosexual and male homosexual transmission accounted for 69.6% and 25.5% of reported infections in 2017, respectively [8].Men who have sex with men (MSM) are at high risk of HIV infection. In Beijing, MSM is the main transmission route [9], with HIV infection rate 100–400 times higher than general population and also higher than drug users (9.08%) and sex workers (0.36%) [10–12]. In addition, concealment of this population makes both its discovery and intervention very difficult. How to improve the detection accessibility of MSM population has become the key to control its HIV infection. Fear of privacy exposure is a major reason of reluctance to obtain HIV face-to-face testing with medical staff. Internet-based Self-sampling at home plus Laboratory testing (ISL) is anonymous and does not have to provide services face to face, and the examiners do not have concerns about privacy exposure, which will become an important means for the general intervention of the high-risk groups of HIV infection in the future. By now, HIV test mode of internet platform plus urine sampling at home plus antibody testing by professional laboratory has achieved phased success in Beijing [13], the urine detection sensitivity is about 90%, and the window period is about one month [14]. Compared with urine antibody testing, nucleic acid commercial testing kits perform better, with a sensitivity of up to 99% [15] and a window period of 6–11 days [16], thus can greatly improve the positive detection rate, especially for the early infected patients pre-HIV seroconversion. Internet-based self-sampling at home plus delivery testing of nucleic acid in laboratory will provide earlier and more accurate testing service for hard-accessible high-risk groups such as MSM. Dried Blood Spots on filter paper (DBS) facilitate preservation of blood total nucleic acids (Total Nucleic Acid, TNA). Characteristics like low biological risk, easy be stored at room temperature within one week with high antibody and nucleic acid stability, make DBSs can be mailed to professional laboratories safely and conveniently [17–19]. In this study, we propose a new mode of HIV test and surveillance among MSM population: Internet-based Self-sampling at home plus Laboratory testing of HIV TNA in DBS (ISL of DBS TNA). Feasibility of ISL of DBS TNA was studied. Characteristics of the new mode and that of traditional surveillance mode at HIV voluntary counseling and testing clinic (VCT) were compared.

## Methods

### Procedures of ISL of DBS TNA

A non-governmental organization (NGO) was entrusted by CDC to release information of DBS nucleic acid testing activity on its WeChat Official Account. The entry conditions were: 18 years or older, have sex with men within last year, work or live in Beijing, and participate in the novel surveillance mode for the first time this year. MSM people interested in the activity should filled in the online questionnaire firstly, and then applied for the DBS self-sampling service package online. The NGO would send out the DBS self-sampling service package according to the address and contact information provided in the questionnaire. Participants should read instructions carefully, watch the self-sampling training video online and then complete DBS self-sampling at home. DBSs should be mailed to the designated address after drying. Designated professional laboratory would perform nucleic acid testing of DBSs received and upload test results to a internet platform. Participants could query the nucleic acid test results online with their unique ID. CDC would follow up participants with positive nucleic acid results using IDs and contact information.

### Protocol of DBS self-sampling at home

Firstly, stretch your arms downward, close hands and shake them hardly back and forth 5–10 times. Relax your wrist and massage the prick site gently to promote blood circulation. If necessary, hot compress can be applied before finger-prick. Wipe the fingertip with 75% alcohol pads and dry naturally. Unscrew the cap of Unistik 3 18G lancet (Owen Mumford). Compact the platform surface of the lancet on the belly sides of middle or index finger. Press the trigger button to prick and then gently, intermittently, and repeatedly apply pressure to the tissue surrounding the prick site. Drop the fingertip blood in the printing rings on the filter paper to make 3 full blood spots. The blood spot cards should be naturally dried at room temperature for 4 h with no direct sunlight shed on. Put each prepared DBS sample into a Ziplock bag containing a humidity indicator card and desiccant and mail it to a professional laboratory.

The used lancet, hemostatic cotton balls and other consumables contaminated with blood can be sprayed with 75% alcohol, or dried at room temperature for 4 h, then be put back into the ziplock bag for consumables and mailed together with the DBS sample bag to professional laboratories.

The private query card in the service package with a unique query ID number and the query website, and should be kept properly.

### Nucleic acid detection method for DBSs

Each whole blood spot was punched off and transferred to a sample tube with a disposable forcep. 1000 μL of DBS pre-extraction reagent (COBAS®AmpliPrep/COBAS®TaqMan®Specimen Pre-Extraction Reagent) was added and incubated in a digital shaking drybath for 10 min with 56 °C at 1000 rpm. Then DBS TNA was extracted using extraction reagent (COBAS®AmpliPrep/COBAS®TaqMan®HIV-1 Qualitative Test, v2.0) on COBAS®AmpliPrep instrument (Roche Diagnostics, Basal, Switzerland) and detected by PCR on a COBAS®TaqMan®48 Analyzer (Roche Diagnostics, Basal, Switzerland). Internal and external controls were set for each run. Positive and negative nucleic acid results were reported as "HIV-1 detected" and "HIV-1 not detected", respectively.

### Procedures of surveillance at VCT

The survey subjects was recruited with entry conditions as follows: 18 years or older, have sex with men within last year, work or live in Beijing, and participate in sentinel monitoring for the first time this year. The respondent-driven sampling (RDS) method was adopted to select charismatic members of the target population as seeds for starting the survey, and no more than three cards were issued for each seed to recruit others to participate in the survey. Each card recruited one person. A face to face questionnaire survey was conducted among the subjects after informed consent was obtained by staff with consulting experience and uniform training in accordance with the principles of voluntariness and confidentiality. At the same time, 3-5 mL venous blood samples were collected to detect HIV antibodies by antibody enzyme linked immunosorbent assay (ELISA) with two antibody screening reagents (Wantai, Beijing, China; Lizhu, Shenzhen, China) in the laboratory. If ELISA screening is positive, HIV antibody Western Blot (WB) test will be continued with HIV antibody confirmation reagent (IMT, Shanghai, China).

### Statistical analysis

Statistical calculations were performed using SPSS 19.0.

## Results

**Fig. 1:**
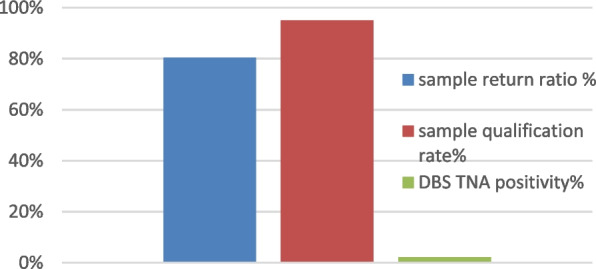
ISL of DBS TNA

For ISL of DBS TNA mode, a total of 423 questionnaires were completed, and 423 testing service packages were sent out; 340 DBSs were mailed to professional laboratories within one month, of which 323 were qualified samples and 17 samples with insufficient blood collection or without penetrating filter paper are considered unqualified; HIV TNA testing of 323 DBSs were completed, and 7 cases were detected positive. Each corresponding percentage is shown in Fig. 1.

For surveillance at sentinel site, 424 questionnaires were completed and 424 venous blood samples were collected for HIV antibody tested, of which 38 were HIV-1 antibody positive.

### Comparison of the composition ratios of the questionnaire population from ISL of DBS TNA and sentinel monitoring at VCT

The questionnaire populations of the two modes were compared using the chi-square test and Fisher's exact test. There was a statistical difference in the overall composition between the population of ISL of DBS TNA and the population monitored at the sentinel sites of VCT (Table 1). Further chi-square analysis showed that ISL of DBS TNA group had a significantly higher proportion of unmarried, company employee/student/official or manager or civil servant, high income group with income > 10,000, sexual orientation of male, no regular sexual partner, previous drug use, HIV testing in the past year, use of post-exposure prophylaxis (PEP), an education level of college or above, and young age (< 40 years old) than sentinel site group (all* P* values < 0.05).

**Table 1:** Comparison of the population composition between ISL of DBS TNA and sentinel site at VCT

Variable	ISL of DBS TNA (*n* = 423)	Sentinel site (*n* = 424)	Chi-square test	
	Number of cases (%)	Number of cases (%)	*χ*^*2*^	*P*
Marital status			-	< 0.001^a^
Married	29 (6.86)	161 (37.97)		
Unmarried	379 (89.6)	199 (46.93)		
Divorced or widowed	15 (3.55)	61 (14.39)		
Lavender marriage	0(0)	3 (0.71)		
Occupation			263.585	< 0.001
Company employee	223 (52.719)	113 (26.651)		
Student	73 (17.258)	2 (0.472)		
Official/manager/civil servant	30 (7.092)	9 (2.123)		
Worker	12 (2.837)	32 (7.547)		
Business services	23 (5.437)	172 (40.566)		
Catering and food industry	8 (1.891)	44 (10.377)		
Others	54 (12.766)	52 (12.264)		
Monthly income			155.172	< 0.001
No income	43 (10.17)	5 (1.18)		
< 2000	15 (3.55)	10 (2.36)		
2000–4000	24 (5.67)	135 (31.84)		
4000–10,000	155 (36.64)	194 (45.75)		
> 10,000	186 (43.97)	80 (18.87)		
Sexual orientation			-	< 0.001^a^
Homosexual	336 (79.43)	285 (67.2)		
Heterosexual	3 (0.71)	1 (0.2)		
Bisexual	84 (19.86)	138 (32.5)		
Sexual roles			6.741	0.034
Receptive	98 (27.22)	87 (22)		
Insertive	130 (36.11)	179 (45.2)		
Both receptive and insertive	132 (36.67)	130 (32.8)		
Blank	63 (14.9)	28 (6.60)		
Regular sexual partner			22.111	< 0.001
Yes	205 (48.46)	225 (65.4)		
No	218 (51.54)	119 (34.6)		
Condom use in anal sex			42.013	< 0.001
Every time	156 (47.42)	207 (60.2)		
Sometimes	158 (48.02)	90 (26.2)		
Never	15 (4.56)	47 (13.7)		
Blank	94 (22.2)	80 (18.9)		
Condom use in oral sex			28.475	< 0.001
Every time	18 (5.29)	40 (9.8)		
Sometimes	75 (22.06)	36 (8.8)		
Never	247 (72.65)	331 (81.3)		
Blank	83 (19.6)	16 (3.77)		
Engagement in commercial homosexual behaviours			6.844	0.009
Yes	15 (3.72)	34 (8)		
No	388 (96.28)	390 (92)		
Blank	20 (4.73)	0(0)		
Previous drug use			231.617	< 0.001
Yes	189 (45.99)	6 (1.4)		
No	222 (54.01)	418 (98.6)		
Blank	12 (2.84)	0(0)		
Have you ever had an STD			24.912	< 0.001
Yes	19 (4.6)	63 (14.9)		
No	394 (95.4)	361 (85.1)		
Blank	10 (2.36)	0(0)		
Have you ever had HIV testing in the past year			46.92	< 0.001
Yes	368 (87)	285 (67.2)		
No	55(13)	139 (32.8)		
Have you ever taken post-exposure prophylaxis				
Yes	32 (9.85)	8 (1.9)	23.056	< 0.001
No	293 (90.15)	416 (98.1)		
Blank	98 (2.32)	0(0)		
The most common places to find sex partners			-	-
Sauna and foot massage	20(-)	5 (1.18)		
Bar, cabaret, tea house, recreation clubs	22(-)	9 (2.12)		
Sports group	19(-)	1 (0.24)		
Referred by a friend	87(-)	43 (10.14)		
Park, public toilet, green space	15(-)	81 (19.1)		
Internet	379(-)	264 (62.26)		
Other	0(-)	21 (4.95)		
Educational level			305.727	< 0.001
Illiterate	2 (0.47)	2 (0.47)		
Primary school	0(0)	33 (7.78)		
Junior high school	8 (1.89)	123 (29.01)		
High school or secondary school	18 (4.26)	111 (26.18)		
College and above	395 (93.38)	155 (36.56)		
Age			-	< 0.001^a^
< 20	4 (0.97)	1 (0.2)		
20–39	380 (91.79)	195 (46)		
≥ 40	30 (7.25)	228 (53.8)		
Blank	9 (2.13)	0 (zero)		

^a^Fisher's exact test

In the ISL of DBS TNA group, the percentages of workers/commercial services/restaurant and food industry workers, bisexual, sexual role as insertive, previous commercial sex behavious, and history of sexually transmitted diseases (STD) were significantly lower than the sentinel site group (all *p*-values < 0.05).

### Differences in HIV prevalence between the populations from ISL of DBS TNA and sentinel monitoring at VCT

The overall HIV prevalence rate was 2.17% and 8.96% in the populations from ISL of DBS TNA and sentinel monitoring at VCT, respectively (*χ*^*2*^ = 14.953, *P* = 0.000 < 0.05) and ISL of DBS TNA had a lower HIV prevalence rate than sentinel site in all categories (Table 2). In ISL of DBS TNA, there was an overall difference in infection rates among the occupational categories (Fisher's exact test, *P* = 0.036), but no significant difference was observed in the pair-wise comparisons; no statistical difference in the overall infection rates was identified in other categories (*P* > 0.05). In sentinel site, differences in the overall distribution of infection rates by marital status, occupation, sexual orientation, sexual role, condom use for anal sex, history of STDs, HIV testing in the past year, and education level were observed; it was found in the risk assessment that men who were divorced or lost their spouse had a 4.314 times greater risk of infection than those that were married (95% CI 1.561–11.921); the infection risk of commercial service providers was 4.853 times that of company employees (95% confidence interval (CI) 1.645–14.311); the risk of infection for those who were homosexual was 2.783 times greater than the risk of bisexuals (95% CI 1.135–6.823); the risk of sexual role as receptive was 3.136 times greater than the risk of insertive (95% CI 1.412–6.968); the risk of those with a previous history of STD was 4.012 times greater than the risk of those with no previous history of STD (95% CI 1.945–8.276); the risk of those with no previous test for HIV in the past year was 5.235 times greater than that of those having been tested for HIV (95% CI 2.552–10.736). Reasons of lower prevalence in ISL of DBS TNA mode might be composition ratio difference compared with traditional sentinel surveillance, but can also be high acceptability rates of the novel mode, which need to be investigated further in the future study.

**Table 2:** Differences in prevalence rates between the two surveillance modes

Variable	ISL of DBS TNA (*n* = 323)		Sentinel sites (*n* = 424)		Chi-square test	
	Number of cases	Number of infections (%)	Number of cases	Number of infections (%)	*χ*^*2*^	*P*
Marital status						
Married	24	0(0)	161	7 (4.35)	-	0.597^a^
Unmarried	287	7 (2.44)	199	21 (10.55)	14.429	0
Divorced or widowed	12	0(0)	61	10 (16.39)	1.104	0.293^b^
Lavender marriage	0	0(-)	3	0(0)	-	-
Occupation						
Company employee	179	2 (1.12)	113	4 (3.54)	0.996	0.318^b^
Student	53	1 (1.89)	2	0(0)	-	1^a^
Official/manager/civil servant	22	0(0)	9	1 (11.11)	-	0.29^a^
Worker	8	1 (12.5)	32	2 (6.25)	-	0.498^a^
Commercial services	15	1 (6.67)	172	26 (15.12)	0.26	0.61^b^
Catering and food industry	5	1(20)	44	3 (6.82)	-	0.359^a^
Other	41	1 (2.44)	52	2 (3.85)	-	-
Monthly income						
No income	31	0(0)	5	0(0)	-	-
< 2000	10	0(0)	10	2 (20.0)	-	0.474^a^
2000–4000	21	2 (9.52)	135	15 (11.11)	0	1^b^
4000–10,000	123	2 (1.63)	194	17 (8.76)	6.805	0.009
> 10,000	138	3 (2.17)	80	4(5)	0.551	0.458^b^
Sexual orientation						
Homosexual	259	7 (2.70)	285	32 (11.23)	14.819	0
Heterosexual	0	0(-)	1	0(0)	-	-
Bisexual	64	0(0)	138	6 (4.35)	1.558	0.212^b^
Sexual role						
Receptive	77	3 (3.90)	87	16 (18.39)	8.378	0.004
Insertive	95	1(1.05)	179	12 (6.7)	3.224	0.073^b^
Both receptive and insertive	102	3 (2.94)	130	10 (7.69)	2.439	0.118
Blank	49	0(0)	28	0(0)		
Regular sexual partner						
Yes	161	2 (1.24)	225	16 (7.11)	7.271	0.007
No	162	5 (3.09)	119	16 (13.45)	10.647	0.001
Blank	0	0(-)	80	6		
Condom use in anal sex						
Every time	120	3 (2.5)	207	20 (9.66)	5.959	0.015
Sometimes	122	3 (2.46)	90	12 (13.33)	9.315	0.002
Never	12	0(0)	47	0(0)	-	-
Blank	69	1	80	6		
Condom use in oral sex						
Every time	15	0(0)	40	7 (17.5)	1.639	0.201^b^
Sometimes	60	0(0)	36	6 (16.67)	8.012	0.005^b^
Never	189	5 (2.65)	331	25 (7.55)	5.329	0.021
Blank	59	2	17			
Has there been commercial homosexual behaviours						
Yes	11	0(0)	34	4 (11.76)	-	0.558^a^
No	299	7 (2.34)	390	34 (8.72)	12.297	0
Blank	13	0(0)	0	0(-)		
Previous drug use						
Yes	144	2 (1.39)	6	2 (33.33)	-	0.008^a^
No	171	5 (2.92)	418	36 (8.61)	6.603	0.014
Blank	8	0(0)	0	0(-)		
Have you ever had an STD						
Yes	14	1 (7.14)	63	14 (22.22)	0.838	0.36^b^
No	307	6 (1.95)	361	24 (6.65)	8.522	0.004
Blank	2	0(0)	0	0(-)		
Have you ever had HIV testing in the past year						
Yes	285	6 (2.11)	285	12 (4.21)	2.065	0.151
No	38	1 (2.63)	139	26 (18.71)	5.964	0.015
Have you ever taken post-exposure prophylaxis						
Yes	21	0(0)	8	1 (12.5)	-	0.276^a^
No	225	6 (2.67)	192	12 (2.88)	0.025	0.873
Blank	77	1 (1.30)	224	25 (11.2)		
Common places to look for sex partners (multiple choice)						
Sauna, bath, and foot massage	15	0(0)	5	0(0)	-	-
Bar, cabaret, tea house, and recreational clubs	19	0(0)	9	0(0)	-	-
Sports group	14	0(0)	1	0(0)	-	-
Referred by a friend	65	2 (3.08)	43	5 (11.63)	1.871	0.171^b^
Park, public toilet, green space	12	0(0)	81	7 (8.64)	-	0.589^a^
Internet or mobile apps	288	7 (2.43)	264	25 (9.47)	12.497	0
Others	0	0(-)	21	1 (4.76)	-	-
Educational level						
Illiteracy	0	0(-)	2	0(0)	-	-
Primary school	0	0(-)	33	6 (18.18)	-	-
Junior high school	3	0(0)	123	5 (4.07)	-	1^a^
High school or secondary school	16	0(0)	111	10 (9.01)	0.569	0.451^b^
College and above	304	7 (2.30)	155	7 (4.52)	1.035	0.309^b^
Age						
< 20	2	1(50)	1	0(0)	-	1^a^
20–39	296	6 (2.03)	195	20 (10.26)	15.875	0
≥ 40	25	0(0)	228	18 (7.89)	1.098	0.295^b^

^a^ Fisher's exact test

^b^ Continuous correction chi-square test

ISL of DBS targets the total nucleic acid of HIV which is detectable 6 to 11 days after exposure, with a much shorter detection window than the one to three months for HIV antibody testing. In ISL of DBS TNA surveillance, 7 samples were found to be positive for nucleic acid, and the nucleic acid testing of the samples were also positive upon repeated testing. The Ct values for the nucleic acid tests were all less than 34, which could be used to determine that the subjects were infected with HIV. Among them, three subjects were exposed 7–10 days prior to testing and six subjects were exposed within seven days to one month. The infected individuals were aged 19–30 years, and all had college education level or above, and one subject was a student. Excluding one blank answer, none of the other 6 infected had received PEP. Five subjects never used condoms during oral sex, and three of them used condoms every time during anal sex, suggesting the necessity of condom use during oral sex in order to prevent infection. Sexually transmitted infections (STIs) were a cofactor of HIV infection [20], and one infected subject had been diagnosed with STD.

### Distribution of unqualified DBSs

A total of 340 DBSs were returned, of which 17 were unqualified samples and 323 were correctly collected samples, with a sample qualification rate as high as 95.0%, which confirmed the high success rate and satisfactory results of self-administered blood collection following the instructions in the service package we designed and the operational video we made. To further improve the correct sample rate, the distribution of unqualified samples was analyzed (Table 3), and no statistical difference was found between the categories (all* P* values > 0.05).

**Table 3:** Distribution of unqualified DBSs

Variable	Number of DBS returned *n* = 340		*χ*^*2*^	*P*
	Number of cases	Number of unqualified samples (%)		
Marital status			-	0.419^a^
Married	24	0 (0.00)		
Unmarried	303	16 (5.28)		
Divorced or widowed	13	1 (7.69)		
Lavender marriage	0	0(-)		
Occupation			-	0.715^a^
Company employee	189	10 (5.29)		
Student	56	3 (5.36)		
Official/manager/civil servant	23	1 (4.35)		
Worker	8	0 (0.00)		
Commercial services	16	1 (6.25)		
Catering and food industry	6	1 (16.67)		
Other	42	1 (2.38)		
Monthly income			-	0.250^a^
No income	34	3 (8.82)		
< 2000	11	1 (9.09)		
2000–4000	22	1 (4.55)		
4000–10,000	126	3 (2.38)		
> 10,000	147	9 (6.12)		
Sexual orientation			-	0.213^a^
Homosexual	275	16 (5.82)		
Heterosexual	0	0(-)		
Bisexual	65	1 (1.54)		
Sexual role			-	0.710^a^
Receptive	79	2 (2.53)		
Insertive	101	6 (5.94)		
Both receptive and insertive	108	6 (5.56)		
Blank	52	3 (5.77)		
Regular sexual partner			0.062	0.803
Yes	170	9 (5.29)		
No	170	8 (4.71)		
Blank	0	0 (-)		
Condom use in anal sex			-	0.592^a^
Every time	126	6 (4.76)		
Sometimes	130	8 (6.15)		
Never	13	1 (7.69)		
Blank	71	2 (2.82)		
Condom use in oral sex			-	0.897^a^
Every time	15	0 (0.00)		
Sometimes	62	2 (3.23)		
Never	201	12 (5.97)		
Blank	62	3 (4.84)		
Has there been commercial homosexual behaviours			-	0.051^a^
Yes	13	2 (15.38)		
No	312	13 (4.17)		
Blank	15	2 (13.33)		
Previous drug use			-	0.578^a^
Yes	154	10 (6.49)		
No	177	7 (3.95)		
Blank	9	0 (0.00)		
Have you ever had an STD				
Yes	16	0 (0.00)	-	1.000^a^
No	316	17 (5.38)		
Blank	8	0 (0.00)		
Have you ever had HIV testing in the past year			1.121	0.290^b^
Yes	298	13 (4.36)		
No	42	4 (9.52)		
Have you ever taken post-exposure prophylaxis			-	0.834^a^
Yes	22	1 (4.55)		
No	236	11 (4.66)		
Blank	82	5 (6.1)		
The most common places to find sex partners (multiple choice)			-	0.904^a^
Sauna, bath, and foot massage	16	1 (6.25)		
Bar, cabaret, tea house, and recreation clubs	20	1(5)		
Sports group	14	0 (0.00)		
Referred by a friend	70	5 (7.14)		
Park, public toilet, green space	13	1 (7.69)		
Internet or mobile app	305	17 (5.57)		
Other	0	0(-)		
Educational level			-	0.151^a^
Illiterate	0	0(-)		
Primary school	0	0(-)		
Junior high school	4	1 (25)		
High school or secondary school	17	1 (5.88)		
College and above	319	15 (4.7)		
Age			-	0.740^a^
< 20	2	0 (0.00)		
20–39	308	17 (5.52)		
≥ 40	25	0 (0.00)		
Black	5	0 (0.00)		

^a^ Fisher's exact test

^b^ Continuous corrected Chi-square test

## Discussion

The purpose of comparing HIV prevalence rates is to reveal the difference in the true infection rates between the two modes. Roche qualitative diagnosis kits with 100% sensitivity and 99.9% specificity were used in our study to detect HIV TNA in DBS specimens, with its’ ellipse period much shorter than window period of antibody detection, thus can provide better performance than antibody test to represent true infection rate. In ISL of DBS TNA mode, assuming HIV TNA positivity representing true infection rate is reasonable. However, as a retrospective study, no paired sentinel DBS samples were collected in the year of 2020, so the corresponding TNA test could not be carried out for sentinel surveillance. In order to prevent undetected infector in sentinel surveillance mode, telephone follow-up and national epidemic database queries were conducted for negative antibody cases with high-risk behaviors. No undetected infectors were found in sentinel surveillance in 2020.

Although HIV prevalence comparison in our study here is not perfect, it can be inferred that the conclusion of lower HIV prevalence in mode of ISL of DBS TNA will not change even when paired sentinel DBSs were collected and TNA tested in the year of 2020. The reason is that TNA test will discover more infections before seroconversion when antibody test failed to find out and thus widen the prevalence gap between ISL of DBS TNA and sentinel surveillance mode. Of course, situation might change if more high-risk population adopted ISL of DBS TNA mode rather than traditional sentinel surveillance at VCT clinic in the future. In the future study, we’ll prepare paired DBSs in sentinel surveilance mode and uncover the true infection rate by HIV TNA.

Compared with the sentinel sites, the population covered by ISL of DBS TNA included more young people with higher education level, higher income, higher proportion of previous use of PEP, higher proportion of HIV testing in the past year, and lower proportion of STDs, indicating that the population using this mode was better educated and more self-protective. However, the high proportion of people without regular sexual partners and the high proportion of previous drug use also suggested high risk of infection in this population. There was an overall difference in the infection by occupation in ISL of DBS TNA (Fisher's exact test, *P* = 0.036), but no significant difference was observed in pair-wise comparisons. This may be due to the low infection rate in ISL of DBS TNA. The surveillance of ISL of DBS TNA should be scaled up to identify the risk factors in those who use this mode.

The 17 unqualified samples were followed up by telephone calls, and in all cases, the lack of success was due to the subjects’ failure to follow the instructions and the sampling training video. This was the case especially in relation to the lack of attention to the pre-sampling steps of warming and massage, which had resulted in insufficient bleeding volume and a sample that could not be analysed. The blood was not able to transmit HIV four hours after drying, and the return rate of contaminated alcohol pads and lancet was 97.1% (330/340), indicating that the possibility of biological contamination of the environment caused by self-administered blood collection was very low. Therefore, when users read the instructions of the service package and watch the sampling training video online, qualification rate of the self-collected samples in the high-risk population was satisfactory and biohazards were low, making DBS self-sampling feasible to be widely promoted in future HIV surveillance testing.

ISL of DBS mode showed that 6.50% (21/323) of the subjects had received PEP for HIV. Among the 21 individuals, 95.2% were aged 21–39 years, and 100% had college education level or above. All of these subjects tested negative for HIV nucleic acid, which preliminarily suggested the effectiveness of PEP in preventing HIV infections [21].

In ISL of DBS mode, 61.9% of the subjects had used novel drugs such as Rush and the proportion of subjects who had received PEP was higher than the sentinel sites, suggesting that such population was more willing to accept new information, which was an important reason for the acceptance of the self-collected blood samples and delivery for testing in this population.

## Conclusions

In conclusion, this study provided a method of DBS preparation through self-sampling among high-risk populations with high qualified sample rate and low environmental biohazard. The method consisted of online questionnaire and offline mailing of the service package. The surveillance route of self-sampling and delivery testing had better confidentiality and covered a population that was a supplement to the sentinel sites. This enabled earlier detection of infected individuals and could be widely promoted in the field of AIDS prevention and treatment.

Further studies are still needed.

## Data Availability

The datasets used or analyzed during the current study are available from the corresponding author on reasonable request.

## Abbreviations

MSM: Men who have sex with men
DBS: Dried blood spot
TNA: Total nucleic acid
ISL: Internet-based self-sampling at home plus laboratory testing
VCT: Voluntary counseling and testing clinic
NGO: Non-governmental organization
CDC: Center for Disease Control and Prevention
RDS: Respondent-driven sampling
ELISA: Enzyme linked immunosorbent assay
WB: Western Blot
PEP: Post-exposure prophylaxis
STD: Sexually transmitted diseases

## References

[1] Wu ZY (2019). HIV/AIDS prevention strategy with Chinese characteristics. Chin J Dis Control Prev.

[2] Cohen MS, Shaw GM, McMichael AJ (2011). Acute HIV-1 infection. N Engl J Med.

[3] Powers KA, Ghani AC, Miller WC (2011). The role of acute and early HIV infection in the spread of HIV-1 in Lilongwe, Malawi: Implications for “test and treat” and other transmission prevention strategies. Lancet.

[4] Brenner BG, Roger M, Routy JP (2007). High rates of forward transmission events after acute/early HIV-1 infection. J Infect Dis.

[5] Robb ML, Eller LA, Kibuuka H (2016). Prospective study of acute HIV-1 infection in adults in east Africa and Thailand. N Engl J Med.

[6] Hollingsworth TD, Anderson RM, Fraser C (2008). HIV-1 transmission, by stage of infection. J Infect Dis.

[7] Li Y, Han Y, Xie J (2014). CRF01_AE subtype is associated with X4 tropism and fast HIV progression in Chinese patients infected through sexual transmission [J]. AIDS.

[8] Transcript of the regular press conference of the National Health Commission of China on November 23, 2018. http://www.nhc.gov.cn/wjw/xwdt/201811/b0ca3817224e44899a3248a0f6e48948.

[9] Cheng JJ, Jin X, Wang C (2021). Bayesian inference of HIV transmission in MSM population in Beijing. J Shanghai Univ (Natural Science Edition).

[10] Zhang L, Chow EP, Jing J (2013). HIV prevalence in China: integration of surveillance data and a systematic review. Lancet Infect Dis.

[11] Lau JTF, Li DL, Wang ZX (2015). Repeated HIV voluntary counseling and testing increased risk behaviors among men who have sex with men in China: a prospective cohort study. AIDS Behav.

[12] Han XX, Xu JJ, Chu ZX (2011). Screening acute HIV infections among chinese men who have sex with men from voluntary counseling & testing centers. PLOS One.

[13] Xia D, Feng X, He X (2018). Feasibility of an internet-based HIV testing service: anonymous urine collection from men who have sex with men. AIDS Care.

[14] Zhang L, Wang XX, Liu ZY, et al. Application of ELISA in detecting HIV-1 antibody in urine. Chin J AIDS STD. 2016;22:1008,1010.

[15] Templer SP, Seiverth B, Baum P (2016). Improved sensitivity of a dual-target HIV-1 qualitative test for plasma and dried blood spots. J Clin Microbiol.

[16] Robe´rioAmorim de Almeida Ponde´ (2011). Genomic detection of human immunodeficiency virus (HIV) by nucleic acid amplification test in a frequent platelet donor during the pre-seroconversion period. Arch Virol.

[17] Fahimah A, Erick A, Yingfen H, et al. Reliability of dried blood spot (DBS) cards in antibody measurement: a systematic review. PLoS One. 2021. https://journals.plos.org/plosone/article?id=10.1371/journal.pone.0248218. 10.1371/journal.pone.0248218 PMC7959368 33720928

[18] Susan CA, Carole LW, Wendy S, et al. Stability of HIV-1 nucleic acids in dried blood spot samples for HIV-1 drug resistance genotyping. PLoS One. 2015. https://journals.plos.org/plosone/article?id=10.1371/journal.pone.0131541. 10.1371/journal.pone.0131541 PMC4493047 26147689

[19] Chaisomchit S, Wichajarn R, Janejai N (2005). Stability of genomic DNA in dried blood spots stored on filter paper. SE Asian J trop Med Pub Hlth.

[20] Xu Y, Wu S, Fu X, et al. Trends in HIV prevalence and HIV-related risk behaviors among male students who have sex with men from 2016 to 2020 in Nanjing, China: consecutive cross-sectional surveys. Front Public Health. 2022;10:806600. https://www.ncbi.nlm.nih.gov/pmc/articles/PMC9091556/pdf/fpubh-10-806600.pdf. 10.3389/fpubh.2022.806600 PMC9091556 35570976

[21] DeHaan E, McGowan JP, Fine SM (2021). New York State Department of Health AIDS Institute Clinical Guidelines: PEP to Prevent HIV Infection.

